# The effect of antibiotic use on prevalence of nosocomial vancomycin-resistant enterococci- an ecologic study

**DOI:** 10.1186/s13756-017-0253-5

**Published:** 2017-09-13

**Authors:** Cornelius Remschmidt, Michael Behnke, Axel Kola, Luis A. Peña Diaz, Anna M. Rohde, Petra Gastmeier, Frank Schwab

**Affiliations:** Institute of Hygiene and Environmental Medicine, Charité-Universitätsmedizin Berlin, Hindenburgdamm 27, 12203 Berlin, Germany

**Keywords:** Vancomycin-resistant enterococcus, Transmission, Antibiotic use, Carbapenems, Glycopeptides

## Abstract

**Background:**

Vancomycin-resistant enterococci (VRE) are among the most common antimicrobial-resistant pathogens causing nosocomial infections. Although antibiotic use has been identified as a risk factor for VRE, it remains unclear which antimicrobial agents particularly facilitate VRE selection. Here, we assessed whether use of specific antimicrobial agents is independently associated with healthcare-associated (HA) VRE rates in a university hospital setting in Berlin, Germany.

**Methods:**

We conducted the study between January 2014 and December 2015 at the Charité-university hospital of Berlin, Germany. From the hospital pharmacy, we extracted data for all antibacterials for systemic use (anatomical therapeutic chemical (ATC)-classification J01) and calculated ward specific antibiotic consumption in defined daily doses (DDDs) per 100 patient-days (PD). We used the microbiology laboratory database to identify all patients with isolation of invasive or non-invasive VRE and calculated HA-VRE incidence as nosocomial VRE-cases per 100 patients and HA-VRE incidence density as nosocomial VRE-cases per 1000 PD. We defined VRE isolates as hospital-acquired if they were identified three days or later after hospital admission and otherwise as community-acquired (CA-VRE). We performed univariable and multivariable regression analyses to estimate the association of the frequency of HA-VRE per month with antibiotic use and other parameters such as length of stay, type of ward or presence of at least one CA-VRE on ward. In a second analysis, we considered only patients with VRE infections.

**Results:**

We included data from 204,054 patients with 948,380 PD from 61 wards. Overall, 1430 VRE-cases were identified of which 409 (28.6%) were considered hospital-acquired (HA). We found that carbapenem use in the current month and prior-month use of glycopeptides increased the risk for HA-VRE by 1% per 1 DDD/100 PD and 3% per 1 DDD/100 PD, respectively. However, when only VRE from clinical samples were considered, only glycopeptide use showed a statistically significant association. In both models, detection of at least one patient with CA-VRE on a ward in the current month significantly increased the risk of HA-VRE, thereby indicating nosocomial spread of VRE.

**Conclusions:**

Our findings suggest that the risk of HA-VRE is associated with specific antimicrobial agents. Prudent use of these antimicrobial agents might reduce nosocomial VRE rates. That appearance of at least one CA-VRE case on the ward increased the risk of HA-VRE detection highlights the importance of strict hand hygiene practices to interrupt person-to-person transmission of VRE.

## Background

Vancomycin-resistant enterococci (VRE) are emerging worldwide and are among the most common antimicrobial-resistant pathogens causing nosocomial infections [1–4]. Since infections with VRE are associated with prolonged in-hospital stay and excess mortality [5, 6], increasing VRE rates pose a serious threat to global health. In fact, the World Health Organization (WHO) judged VRE to be of high importance in the “Global Priority list of antibiotic-resistant bacteria to guide research, discovery and development of new antibiotics” [7].

So far, several risk factors for VRE colonization or infection have been identified, such as long periods of hospitalization, immunosuppression, serious comorbid conditions, close proximity to patients infected or colonized with VRE as well as antibiotic use [2, 8–12]. Although antibiotic use has been advocated as a major modifiable risk factor [8, 13], controversies remain regarding the impact of specific antimicrobial agents or antimicrobial groups on the selection process of VRE [8, 12].

Therefore, the primary objective of the study was to investigate whether use of specific antimicrobial agents is independently associated with nosocomial VRE rates in a university hospital setting in Berlin, Germany. Secondary objective was to identify other risk factors that are associated with nosocomial VRE rates.

## Methods

This study was conducted at the Charité-university hospital of Berlin, between January 2014 and December 2015. The Charité is a tertiary care center with more than 3000 beds and more than 140,000 inpatient cases per year.

From the microbiology laboratory database of Charité we prospectively identified patients with vancomycin-resistant Enterococcus (*E*.) *faecium-* or *E. faecalis*-isolates from intensive care units (ICUs) and surgical, medical and hemato-oncological wards. In case of screening samples, selective chromogenic medium (chromID® VRE, bioMérieux) and VITEK 2 system (bioMérieux) were used, while clinical samples were processed following standard procedures dependent on specimen type. Results were interpreted according to European Committee on Antimicrobial Susceptibility Testing definitions (EUCAST, http://www.eucast.org). A molecular characterization of the VRE isolates was not performed.

We defined VRE isolates as hospital-acquired (HA-VRE) if they were identified three days or later after hospital admission and otherwise as community-acquired (CA-VRE). In patients with multiple VRE isolates during the same hospital stay, only the first identified isolate was considered. We included isolates irrespective whether they were obtained for infection control surveillance or recovered from clinical specimen and irrespective whether they were associated with colonization or infection.

### Performance of screening

At the Charité, a targeted risk adjusted screening policy is implemented: in patients in whom a VRE has been isolated within the previous three years, a rectal swab is obtained at admission. However, during the study period eight (non-ICU) wards performed an intensified admission screening that involved all patients irrespective of underlying risk factors. In this ecologic study, we did not collect data on compliance with the screening policy or compliance with hand hygiene or cleaning procedures on the respective wards.

### Systemic antibiotic use

Antimicrobial agents were classified according to the anatomical therapeutic chemical (ATC) system (34). From the pharmacy of Charité, ward specific data on antibiotic consumption were converted into defined daily doses (DDDs) and reported as DDD/100 patient-days (PD) at monthly intervals. We collected data for all antibacterials for systemic use (ATC-code J01). Data on antifungal or antiviral medication were not considered.

### Ethics and data protection

For this ecologic study, we analyzed aggregated and anonymous data that were collected by the hospital in accordance with the German “Protection against Infection Act”, §23. Therefore, ethical approval and informed consent were not required and institutional review boards were not consulted.

### Statistical analysis

We calculated median and interquartile range (IQR) for the total study period (24 months) for the following variables: number of patients, patient-days (PD), average length of stay (LOS), number of beds per ward, bed occupancy rate, month of VRE-detection, HA-VRE rates and antibiotic use. HA-VRE incidence was calculated as nosocomial VRE-cases per 100 patients and HA-VRE incidence density as nosocomial VRE-cases per 1000 PD. We reported data for all wards as well as stratified by type of ward (i.e. surgical, medical, ICU, hemato-oncological). Differences in baseline characteristics between different types of wards were compared by using the Kruskal-Wallis test. Univariable and multivariable regression using generalized linear models was performed to estimate the association of the frequency of HA-VRE per month with different antimicrobial groups in the current month and the month before the current month and further confounding parameters such as length of stay (LOS), bed occupancy (patient days/bed places on ward available), type of ward and at least one patient with community-acquired VRE on ward in the current month. In addition, we added a variable that took the different screening policies (screening of all patients on admission irrespective of underlying risk factors on eight wards) into account. Since observations within a ward are not statistically independent due to the diagnostic and management policies (particularly the frequency of microbiological tests and screening), adjusted incidence rate ratios (IRR) with 95% confidence intervals (CI) were estimated. They were based on generalized estimating equation (GEE) models which account for this clustering effect by using an exchangeable correlation structure (35).

We used negative-binomial distribution instead of Poisson distribution because the variance exceeds the mean and overdispersion was observed. The Lagrange multiplier test was used to test whether the negative binomial model significantly differs from the Poisson model. The log number of patient days during each month was used as an offset in the model. All parameter with *p* < 0.2 in the univariable regression model were included in a full model and then non-significant parameters were excluded stepwise. The selection criterion was the smallest Chi-square value and *p* ≥ 0.05 in the type III score statistic. The quasi-likelihood information criterion (QIC) as a modification of the Akaike information criterion (AIC) was used as goodness-of-fit measure in the GEE model. We calculated two models. The first model considered all identified HA-VRE isolates (clinical and screening samples) the second model considered only clinical specimen. Antimicrobial agents that are relevant for the treatment of VRE, such as daptomycin, linezolid and tigecyclin were excluded from the analysis. Additionally, spearman correlation coefficients were calculated between the antibiotic use and the incidence density of HA-VRE for the entire study period (24 months). *P*-values less than 0.05 were considered significant. All analyses were performed using SPSS [IBM SPSS statistics, Somer, NY, USA] and SAS 9.4 [SAS Institute, Cary, NC, USA].

## Results

From 61 wards (20 surgical, 18 medical, 14 ICUs, 9 hemato-oncological) we included data from 204,054 patients with 948,380 patient-days. Median length of stay was 4.7 days (IQR, 3.9–5.8) and was statistically significant higher in ICUs and hemato-oncological wards as compared to surgical wards (see Table 1).

**Table 1:** Descriptive data, VRE cases and antibiotic use of the 61 wards considered in the analysis in total and by type of ward

Characteristic	Type of hospital ward				
Baseline characteristics	All (*n* = 61) Median (IQR)	Surgical (*n* = 20) N (%)/median (IQR)	Medical (*n* = 18) N (%)/median (IQR)	Intensive Care Unit (*n* = 14) N (%)/median (IQR)	Hemato-oncological (*n* = 9) N (%)/median (IQR)
Patients, *N* = 204,054	3260 (1886–4478)	4180 (2642–5002)	3849 (3486–4909)	1483 (1252–1899)	2031 (1576–2268)
Patient-days, *N* = 948,380	14,773 (10,642–19,154)	16,488 (13,488–19,819)	17,912 (15,018–20,758)	8949 (6961–10,642)	13,722 (10,849–14,430)
Average length of stay, d	4.7 (3.9–5.8)	4.4 (3.7–5.0)	4.3 (3.7–5.1)	5.6 (5.1–7.0)	6.4 (4.8–8.2)
Beds, *N* = 1604	27 (18–32)	32 (30–33)	31.5 (26–36)	13 (10–16)	21 (18–21)
Bed occupancy rate (bed-days/patient-days*100)	81.1 (77.4–92.6)	77.2 (67.0–81.5)	79.1 (78.0–82.4)	94.9 (94.0–97.2)	89.5 (81.1–93.1)
VRE rates					
VRE cases (all^a^), *N* = 1430	12 (5–24)	5.5 (4.5–12.5)	7 (3–15)	20 (13–70)	48 (33–69)
Screening cases, *N* = 939 (65.7%)	4 (2–12)	3.5 (1.5–5)	3.5 (2–4)	9 (0–26)	42 (31–63)
Clinical cases, *N* = 491 (34.3%)	4 (2–10)	4 (1.5–6.5)	3 (1–8)	12.5 (8–23)	3 (2–6)
VRE cases (nosocomial), *N* = 409 (28.6%)	3 (1–7)	2 (1–4)	1 (0–4)	11.5 (5–21)	5 (3–8)
VRE incidence (nosocomial VRE/100 patients)	0.11 (0.03–0.32)	0.05 (0.02–0.14)	0.03 (0–0.11)	0.68 (0.32–1.22)	0.29 (0.14–0.53)
VRE incidence density (nosocomial VRE/ 1000 patient-days)	0.24 (0.07–0.54)	0.11 (0.06–0.27)	0.07 (0–0.22)	1.17 (0.71–1.75)	0.35 (0.24–0.54)
Systemic antibiotic use (ATC-code) in DDD/100 patient-days					
All antibacterials for systemic use (J01)	76.8 (61.6–131.9)	72.8 (58.1–83.6)	64.1 (39.2–72.8)	163.2 (144.1–200.7)	115.0 (65.4–123.8)
- Beta-lactam antibacterials, penicillins (J01C)	19.5 (13.8–34.4)	15.6 (12.1–38.7)	17.0 (11.5–28.6)	34.1 (30.1–46.2)	17.6 (16.1–20.4)
- Combinations of penicillins, incl. Beta-lactamase inhibitors (J01CR)	14.2 (10.6–26.8)	12.2 (9.5–34.3)	10.7 (8.4–16.4)	21.4 (15.9–31.0)	15.2 (13.7–18.3)
- Ampicillin and enzyme inhibitor (J01CR01)	5.3 (2.5–16.5)	5.7 (2.8–25.4)	3.8 (2.4–9.7)	11.2 (6.5–17.9)	2.3 (1.0–3.8)
- Piperacillin and enzyme inhibitor (J01CR05)	6.0 (2.6–9.2)	2.7 (1.6–4.1)	3.1 (2.0–5.8)	10.2 (7.2–12.9)	10.4 (7.4–11.5)
- Other beta-lactam antibacterials (J01D)	21.6 (16.9–37.5)	18.6 (14.2–29.2)	18.5 (10.3–22.8)	57.5 (45.7–68.5)	20.5 (18.2–34.5)
- Second-generation cephalosporins (J01 DC)	4 (2.6–11.7)	9.0 (3.4–20.0)	3.3 (1.9–8.2)	4.3 (2.7–17.1)	2.8 (2.1–3.5)
- Third-generation cephalosporins (J01DD)	3.9 (2.2–6.7)	2.4 (1.2–3.4)	3.1 (2.0–6.4)	9.0 (6.6–12.6)	4.3 (3.5–6.3)
- Ceftriaxone (J01DD04)	2.6 (1.2–4.7)	1.6 (0.7–2.8)	2.8 (1.5–5.6)	4.7 (2.8–5.8)	1.6 (0.9–2.8)
- Carbapenems (J01DH)	8.4 (2.3–19.0)	4.6 (1.0–7.5)	3.2 (1.5–8.4)	42.3 (22.5–49.4)	11.8 (10.7–25.7)
- Meropenem (J01DH02)	7.8 (2.2–16.8)	3.3 (0.8–6.8)	2.5 (1.5–7.8)	40.3 (22.1–45.0)	11.5 (10.4–25.4)
- Sulfonamides and Trimethoprim (J01E)	2.7 (1.4–5.0)	2.7 (1.4–3.3)	1.1 (0.6–2.0)	4.3 (3.1–7.1)	13.5 (2.7–15.8)
- Makrolides, lincosamides and streptogramins (J01F)	7.3 (4.5–9.4)	6.4 (3.4–8.4)	6.2 (4.2–7.8)	8.8 (6.4–10.5)	8.0 (7.0–10.4)
- Aminoglycosides (J01G)	0.4 (0.2–1.3)	0.2 (0.1–0.4)	0.2 (0.1–0.6)	3.5 (1.4–7.9)	0.3 (0.2–0.4)
- Quinolones (J01M)	11.2 (6.8–20.7)	8.3 (6.1–16.0)	7.4 (3.7–12.4)	23.3 (17.9–27.3)	13.5 (10.2–43.6)
- Ciprofloxacin (J01MA02)	10.5 (6.0–19.0)	8.0 (5.7–14.1)	6.9 (3.1–11.0)	21.6 (17.4–25.2)	11.1 (9.2–41.9)
- Other antibacterials (J01X)	7.5 (3.5–12.9)	6.1 (3.2–9.3)	3.7 (1.7–6.3)	29.2 (20.8–36.7)	8.8 (5.1–11.0)
- Glycopeptide antibacterials (J01XA)	3.0 (1.5–8.7)	1.8 (1.4–3.1)	1.7 (0.8–2.2)	12.8 (8.7–19.0)	5.6 (3.7–9.4)
- Vancomycin (J01XA01)	2.6 (1.5–6.7)	1.8 (1.4–3.1)	1.6 (0.8–2.2)	12.4 (8.7–16.6)	2.8 (2.6–4.0)

IQR, interquartile range; VRE, vancomycin-resistant enterococci; ATC, Anatomical Therapeutic Chemical classification system

^a^nosocomial and community acquired VRE

### Rates of HA-VRE

Overall, 1430 VRE-cases were identified of which 409 (28.6%) were considered hospital-acquired. Of those, 238 (58.2%) HA-VRE were identified in 2015. The median HA-VRE incidence and HA-VRE incidence density in all 61 wards was 0.11 (IQR, 0.03–0.32) and 0.24 (0.07–0.54), respectively (Table 1); however, rates of HA-VRE differed significantly between types of ward. Highest HA-VRE rates were observed on ICUs (incidence density: 1.17 (0.71–1.75)) and lowest HA-VRE rates on medical wards (incidence density: 0.07 (0–0.22)).

### Systemic antibiotic use

Between 2014 and 2015 median antibiotic use among all wards was 76.8 (IQR, 61.6–131.9) DDD/100 PD. Antibiotic use ranged from 64.1 DDD/100 PD on medical wards to 163.2 DDD/100 PD on ICUs (see Table 1). Particularly, the use of broad-spectrum antibiotics such as third-generation cephalosporins, glycopeptides and carbapenems was 3 to 13-fold higher on ICUs as compared to medical or surgical wards. For example, median carbapenem use on medical wards was 3.2 (1.5–8.4) DDD/100 PD, whereas it was 42.3 (22.5–49.4) DDD/100 PD on ICUs.

### Associations between HA-VRE and antibiotic use

**Fig. 1:**
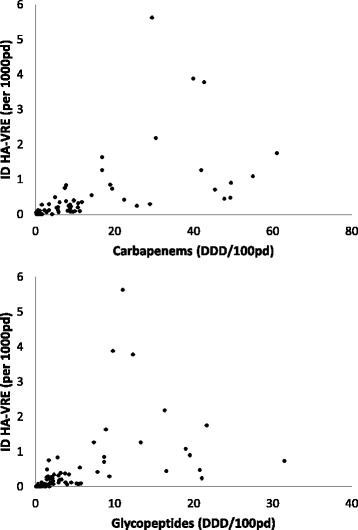
Association between incidence density of hospital-acquired vancomycin resistant enterococci (ID HA-VRE) and antibiotic use density (DDD/100pd) of carbapenems (ATC-code J01DH, Spearman correlation coefficient *r* = 0.801, *p* < 0.001) and glycopeptides (ATC code J01XA, *r* = 0.76, p < 0.001) on 61 wards, 2014–2015, Berlin, Germany

According to Spearmans correlation, several antimicrobial groups were associated with HA-VRE incidence density. For example, carbapenem use (*r* = 0.801, *p* < 0.001, ATC code J01DH), glycopeptide use (*r* = 0.76, p < 0.001, ATC code J01XA) and use of third-generation cephalosporines (*r* = 0.57, p < 0.001, ATC code J01DD) showed a strong correlation with HA-VRE incidence density (see Fig. 1).

The first multivariable regression model (screening and clinical specimen) indicated that carbapenem use in the current month independently increased the risk for HA-VRE by 1% per 1 DDD/100 patient-days (incidence rate ratio (IRR), 1.01; 95% confidence interval (CI) 1.00–1.02, *p* < 0.01), see Table 2. Glycopeptide use in the previous month but not in the current month was also associated with an increased risk of HA-VRE (IRR 1.03; 95% CI, 1.01–1.05, *p* < 0.01). In addition, type of hospital ward (ICU and hemato-oncological ward vs. surgical ward), detection of at least one patient with CA-VRE in the current month, an intensified screening policy and the year 2015 were independently associated with higher VRE rates.

**Table 2:** (including HA-VRE from screening and clinical specimen). Results of multivariable analysis for hospital-acquired VRE cases with antibiotic use by antibiotic group (ATC-code 5-digit)

Parameter/category	IRR	95% CI	*p*-value
Ward			
Medical ward	1 = reference		
Hematological/oncological ward	4.90	1.8–13.3	0.002
Intensive care unit	5.97	2.83–12.60	<.0001
Surgical ward	1.25	0.59–2.63	0.554
At least one patient with community-acquired VRE on ward in the current month	1.69	1.32–2.16	<.0001
Screening of all patients on admission irrespective of underlying risk factors (1 = yes)	2.27	1.01–5.08	0.047
Carbapenem (J01DH) use in the current month (per 1 DDD/100 patient-days)	1.01	1.00–1.02	0.01
Glycopetide (J01XA) use in the previous month (per 1 DDD/100 patient-days)	1.02	1.01–1.04	0.002
Year 2015 (compared to 2014)	1.45	1.17–1.79	<.0001

*DDD* Defined daily dose; *IRR* Incidence rate ratio; *CI* Confidence interval

The second multivariable regression model considering only VRE from clinical specimen showed that glycopeptide use in the previous month (IRR 1.03, 95% CI 1.01–1.05, p < 0.01) were the only antimicrobial agent that was statistically significant associated with HA-VRE (see Table 3). Furthermore, being hospitalized on an ICU, detection of at least one patient with CA-VRE in the current month and the year 2015 increased the risk for HA-VRE.

**Table 3:** (including only HA-VRE from clinical specimen). Results of multivariable analysis for the frequency of hospital-acquired vancomycin resistant enterococci cases with antibiotic use by antibiotic group (ATC-code 5-digit)

Parameter/category	IRR	95% CI	*p*-value
Ward			
Medical ward	1 = reference		
Hematological/oncological ward	1.38	0–60-3.19	0.451
Intensive care unit	4.7	2.02–10.96	<.0001
Surgical ward	1.24	0.55–2.83	0.594
Patient with community-acquired VRE on ward in the current month	1.77	1.19–2.63	0.005
Glycopetide (J01XA) use in the previous month (per 1 DDD/100 patient-days)	1.03	1.01–1.05	0.0038
Year 2015 (compared to 2014)	1.40	1.05–1.86	0.005

*DDD* Defined daily dose; *IRR* Incidence rate ratio; *CI* Confidence interval

In both multivariable models, size of ward, length of stay and bed occupancy rate were no independent risk factors for HA-VRE in the final models.

## Discussion

Antibiotic use is one of the most important risk factors for VRE occurrence within the hospital setting [13]. Although specific antimicrobial agents such as piperacillin/tazobactam [14] or ceftriaxone [15] or antimicrobial groups such as glycopeptides, third-generation cephalosporines and fluoroquinolones [16–18] have been found to be associated with increasing VRE prevalence, controversies remain. For example, although a large prospective study found that vancomycin was strongly associated with increased prevalence of VRE [18], a systematic review that assessed the impact of reducing vancomycin use on the prevalence and incidence of VRE colonization in the US did not find a positive effect of such interventions [19]. Interestingly, Kritsotakis et al. found that the effect of antibiotic use might influence VRE rates with a time lag, ranging from 2 month for vancomycin to 6 months for third-generation cephalosporins. Studies that did not account statistically for such a time delay might therefore miss a possible association.

It is unclear, why among antimicrobial groups only glycopeptides but not carbapenems were associated in both models with HA-VRE rates. A possible explanation is that glycopeptide use constitutes only a first step in the selection process of VRE: Although glycopeptide exposure does not promote emergence of VRE through genetic mutations in individual patients, it might facilitate selection of intestinal VRE [8, 13]. In a second step, use of specific antimicrobial agents further influences the microbial balance leading to high-density colonization with VRE at detectable levels [2, 8, 20].

In fact, Donskey et al. showed that in patients colonized with VRE exposure to antimicrobial agents with activity against Gram-negative bacteria or with anti-anaerobic activity leads to a rapid increase of intestinal VRE density, whereas discontinuation of these antibiotics decreased VRE density [21]. Since fecal density of VRE directly affects the sensitivity of screening results, active surveillance (i.e. rectal screening) for VRE at hospital admission might lead to false-negative results in patients with low VRE densities [22, 23]. In case that VRE-related precaution measures are not implemented, this may contribute to increased spread of VRE within hospitals, particularly if antibiotic therapy is initiated [21]. However, for clinical infections other factors such as immunosuppression or hygiene practices might be more important, factors that were only indirect (ward type ICU; presence of at least one CA-VRE on ward) assessed in our study.

It is also unclear, why carbapenem use in the current but not in the prior month was associated with HA-VRE-rates when screening and clinical specimen were considered. However, given the available evidence it seems unlikely that a single antimicrobial agent is responsible for the multifactorial process of VRE selection. It is rather an interaction of different antimicrobial agents with the competing intestinal microbiota and might differ from individual to individual [8].

Regarding antibiotics, the use of broad-spectrum antibiotics such as glycopeptides and carbapenems was 3 to 13-fold higher on ICUs as compared to medical or surgical wards. Although interpreting these results without individual patient data is challenging, this rates might indicate inappropriate use of these antibiotics on ICUs. Antibiotic stewardship could limit unnecessary or inappropriate use of antibiotics, thereby improving the quality of antibiotic use in hospitals [24].

In addition to glycopeptides and carbapenems, we found that hospitalization on an ICU as well as detection of CA-VRE increased the risk of HA-VRE acquisition. These results are in line with other studies that found that hospitalization on ICUs or severe underlying comorbidities and immunosuppression are risk factors for VRE colonization or infection [2, 12, 25]. That appearance of at least one CA-VRE case on the ward almost doubled the risk of HA-VRE detection highlights the importance of strict hand hygiene practices for health care workers to interrupt person-to-person transmission [26–28].

The fact that HA-VRE cases increased from 2014 to 2015 might indicate an epidemiological trend that has been already observed in other studies [16, 17, 29, 30]; however, the situation regarding spread of VRE in Europe is diverse [1]. Since data in our study were collected for only two years, these results have to be interpreted with caution.

Our study has several limitations. First, we used an ecological study design, which can give valuable insights in understanding the association between antibiotic use and VRE selection; however, causality cannot be proven. Second, we analyzed aggregated data on ward level and had no data on individual patients. Therefore, controlling for important confounders, such as duration of antibiotic therapy, history of antibiotic exposure or severity of underlying illness was not possible. Since it has been shown that group-level and individual-patient-level analyses might yield different results when the association between antibiotic exposure and resistance is assessed [31], our results have to be interpreted with caution. Third, routine screening for VRE at hospital admission was implemented only on some wards at Charité. Patients from other wards may have been colonized on admission or may have acquired VRE in outside hospitals. Therefore, the proportion of HA-VRE might be overestimated [32]. Fourth, the DDD is the assumed average maintenance dose per day for a drug used for its main indication in adults and does not necessarily reflect the recommended or prescribed daily dose [33]. Fifth, a molecular characterization of the identified VRE was not performed and we could not exclude clonal dissemination on the respective wards; however, since we did not identify VRE cluster on ward level during the study period, large outbreaks seems unlikely. Finally, data on compliance with hand hygiene and cleaning procedures were not measured, therefore confounding effects of these factors on our results are unknown.

## Conclusions

In conclusion, we found a positive correlation between prior-month use of glycopeptides and current use of carbapenems and the incidence of HA-VRE on ward level when invasive and non-invasive VRE were considered. In addition, detection of at least one patient with CA-VRE in the current month significantly increased the risk of nosocomial acquisition of VRE, thereby indicating the risk of person-to-person transmission of VRE. To this end, a multifaceted approach to lower HA-VRE rates is required including prudent use of antimicrobial agents as well as implementation of and strict compliance to infection control measures to prevent VRE transmission.

## Abbreviations

95% CI: 95% confidence interval
ATC: Anatomical therapeutic chemical (classification system)
CA: Community-acquired
DDD: Defined daily dose
HA: Hospital-acquired
ICU: Intensive care unit
IQR: Interquartile range
IRR: Incidence rate ratio
LOS: Length of stay
PD: Patient-days
VRE: Vancomycin-resistant enterococcus
